# Nucleation, aggregative growth and detachment of metal nanoparticles during electrodeposition at electrode surfaces†
†Electronic supplementary information (ESI) available: S1 Scharifker–Hills model, S2 tapping mode-atomic force microscopy (TM-AFM) image of AM grade HOPG, after exposure to a droplet of 50 mM KNO_3_, S3 distribution of induction times, S4 results of the modified Cottrell fits at different potentials, S5 FE-SEM images of HOPG after control tip breaking, S6 extended current–time trace. See DOI: 10.1039/c4sc02792b



**DOI:** 10.1039/c4sc02792b

**Published:** 2014-11-07

**Authors:** Stanley C. S. Lai, Robert A. Lazenby, Paul M. Kirkman, Patrick R. Unwin

**Affiliations:** a Department of Chemistry , University of Warwick , Gibbet Hill Road , Coventry CV4 7AL , UK . Email: P.R.Unwin@warwick.ac.uk; b MESA+ Institute for Nanotechnology , University of Twente , PO Box 217 , 7500 AE Enschede , The Netherlands . Email: s.c.s.lai@utwente.nl

## Abstract

The nucleation and growth of metal nanoparticles (NPs) on surfaces is of considerable interest with regard to creating functional interfaces with myriad applications. Yet, key features of these processes remain elusive and are undergoing revision. Here, the mechanism of the electrodeposition of silver on basal plane highly oriented pyrolytic graphite (HOPG) is investigated as a model system at a wide range of length scales, spanning electrochemical measurements from the macroscale to the nanoscale using scanning electrochemical cell microscopy (SECCM), a pipette-based approach. The macroscale measurements show that the nucleation process cannot be modelled as either truly instantaneous or progressive, and that step edge sites of HOPG do not play a dominant role in nucleation events compared to the HOPG basal plane, as has been widely proposed. Moreover, nucleation numbers extracted from electrochemical analysis do not match those determined by atomic force microscopy (AFM). The high time and spatial resolution of the nanoscale pipette set-up reveals individual nucleation and growth events at the graphite basal surface that are resolved and analysed in detail. Based on these results, corroborated with complementary microscopy measurements, we propose that a nucleation-aggregative growth-detachment mechanism is an important feature of the electrodeposition of silver NPs on HOPG. These findings have major implications for NP electrodeposition and for understanding electrochemical processes at graphitic materials generally.

## Abstract 2

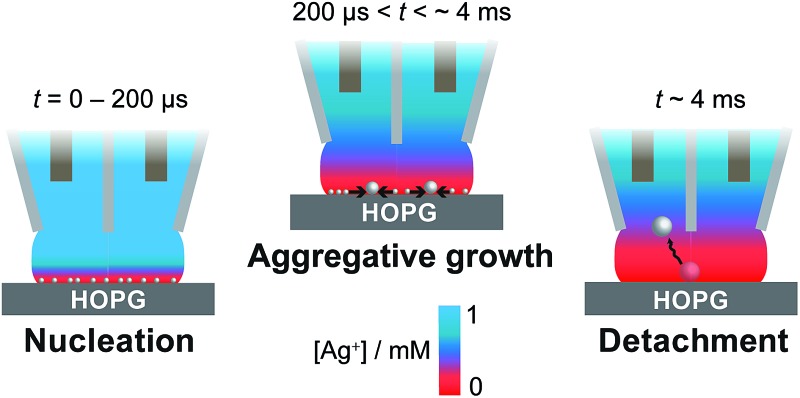
A nucleation-aggregative growth-detachment mechanism is proposed as an important feature of the electrodeposition of silver nanoparticles on basal plane highly oriented pyrolytic graphite (HOPG).

## Introduction

The properties of metal nanoparticles (NPs) can differ significantly from their bulk analogues, and are even tuneable through the size and shape of the particle. Examples of properties that depend on NP size and shape include interatomic bond distances,^1,2^ melting point,^1,3^ chemical reactivity^4–6^ and optical and electronic properties.^1,7,8^ This powerful control over the fundamental properties has given rise to a huge variety of applications of metal NPs, including in sensing,^9,10^ spectroscopy,^10–12^ catalysis,^5,13,14^ as optical filters,^15^ and in biomedical^16–18^ and antimicrobial applications.^19,20^ In many of these applications, NPs are often dispersed as arrays on a support material.

A wide variety of techniques can be used to prepare supported NPs.^21–30^ For conducting supports, electrodeposition is particularly attractive as it allows the direct growth of NPs on a substrate, thereby ensuring electrical connection between the substrate and the NPs. Furthermore, it circumvents the need for NP stabilizing surfactants, which may impact NP reactivity.^13^ Electrodeposition can offer control over the size- and shape-distribution, as well as the spatial distribution of NPs, by tuning the deposition parameters and electrolyte composition.^29,31^

Analysis of the current–time–voltage characteristics during deposition can reveal insights into the electrodeposition mechanism.^30,32–34^ However, such studies typically involve the deposition of large numbers of NPs, with the macroscopic current–time–voltage characteristics fitted with continuous mathematical models to extract nanoscale mechanistic information on the elementary processes involved in NP electronucleation. Interpretation of such data is further complicated by overlapping diffusion fields of neighbouring NPs,^35^ surface-mediated Ostwald ripening,^36,37^ and heterogeneities in substrate properties (*e.g.* different types of nucleation sites).^28^ Whilst there are some reports of the electrodeposition of one or a few NPs (<10),^33,38–40^ they have usually required the use of nanoscale electrodes to restrict the number of nucleation sites, but such electrodes are non-trivial to fabricate and fully characterise.^40,41^ Additionally, the need to encapsulate electrodes in an insulating support severely restricts the range of electrode materials and surface preparations that can be used.

Here, we examine the electrodeposition of silver on (the basal surface of) highly oriented pyrolytic graphite (HOPG) at both the macroscale and nanoscale, making use of scanning electrochemical cell microscopy (SECCM)^42,43^ as a nanoscopic electrochemical cell. This system is particularly interesting for a number of reasons. First, silver deposition on HOPG is often studied as a model system for metal deposition on carbon electrodes, displaying fast heterogeneous electron transfer kinetics.^44–48^ However, previous studies indicate that this process cannot be modelled satisfactorily by the models for either instantaneous or progressive nucleation and growth, instead seemingly showing a behaviour which is intermediate between the two limiting cases.^45,49,50^ Evidently, new insights would be hugely valuable for furthering understanding. Second, the active sites for metal electrodeposition on HOPG have been a topic of debate.^28,44,45,47,51^ For example, the commonly accepted model is that electronucleation occurs solely on step edge and defect sites, with the basal plane being inert. However, in light of recent findings on HOPG basal plane reactivity for other electrochemical processes,^48,52–61^ it is timely and important to readdress this model for metal electrodeposition. Specifically, the macroscale experiments we describe are affected by *both basal and step edge sites*, and by comparing samples of different step edge density (varied by more than two orders of magnitude herein) we are able to explore the contribution of step edges towards the nucleation of NPs. For the nanoscale experiments, given the spatial resolution of SECCM (a few hundred nm), we have a platform to study the intrinsic activity of the *basal plane alone* towards NP deposition without any influence from step edges. Third, a further discrepancy can be found in the apparent density of nuclei, inferred by electrochemistry and measured by microscopy. *Ex situ* characterisation typically shows a nuclei number density up to a few orders of magnitude higher than that obtained by modelling the current–time response.^44,62^ Finally, the ability to cleave and characterise HOPG^54^ offers a clean and reproducible surface characterised by low background currents, thus allowing dynamic measurements to be performed with good time resolution and an appreciable signal-to-noise ratio.

## Experimental

### Materials

**Fig. 1:**
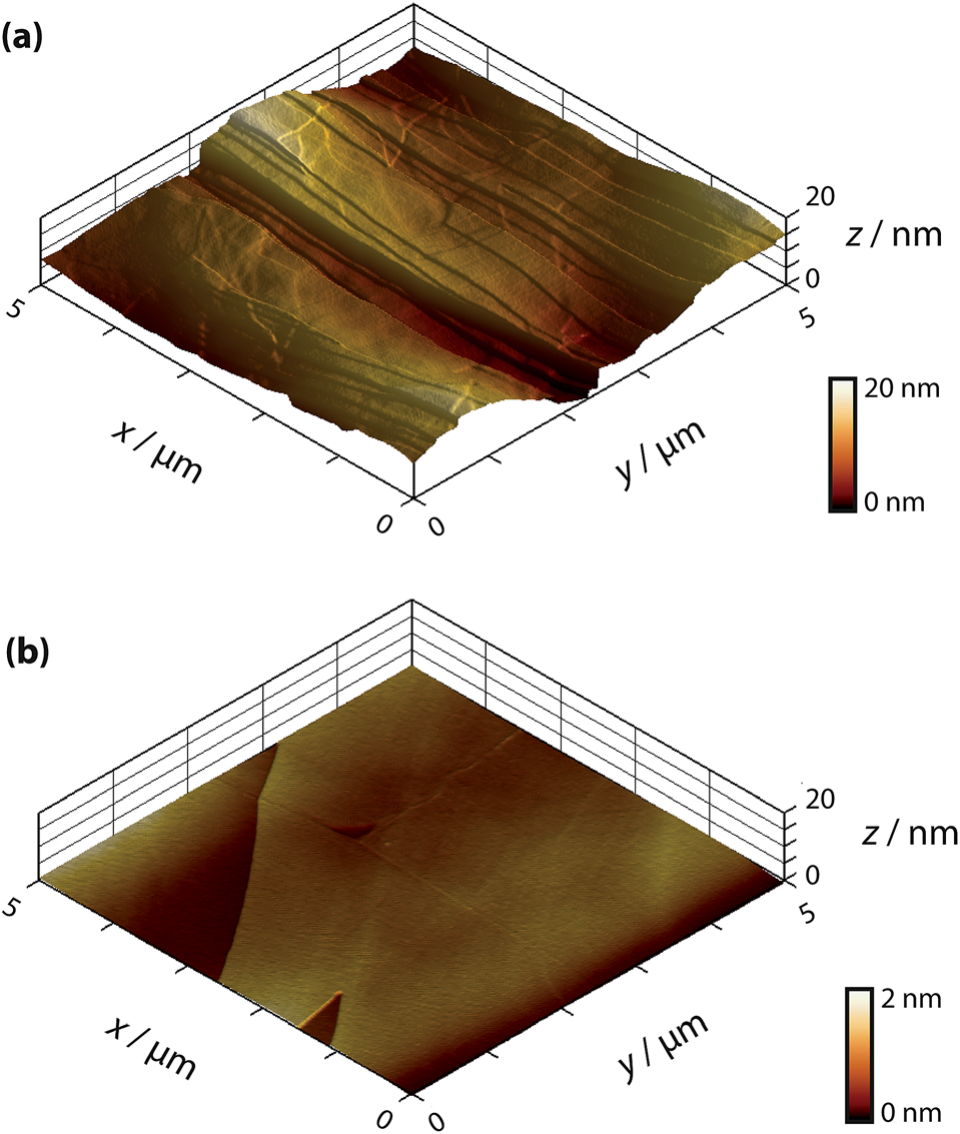
AFM images of freshly cleaved (a) SPI-3 HOPG and (b) AM grade HOPG .

Two grades of HOPG were employed in this study: a high-quality (but ungraded) sample originating from Dr A. Moore (Union Carbide, now GE Advanced Ceramics), hereafter denoted as AM grade HOPG, kindly provided by Prof. R.L. McCreery (University of Alberta), and an SPI-3 graded sample from SPI Supplies (Aztech Trading, UK). Both HOPG samples were freshly cleaved with adhesive tape before each experiment. Previously, we have shown that AM grade HOPG provides surfaces with extensive basal terraces (typically ≫1 μm) and a low coverage of step edges (0.09% area on average, with respect to the basal surface, mostly of mono-atomic height),^54^ whereas SPI-3 has an average step edge coverage of 31% (mostly multilayer steps),^63^ a difference of more than two orders of magnitude in step coverage. Typical atomic force microscopy (AFM) images of these two different surfaces are shown in Fig. 1.

Solutions were prepared from silver nitrate (AgNO_3_, Sigma Aldrich, “ACS reagent”) with potassium nitrate (KNO_3_, Sigma Aldrich, “ReagentPlus”) as supporting electrolyte (except in specific cases noted below) in ultra-pure water (“Select HP”, Purite, >18 MΩ cm at 25 °C). All materials were used as received, and the concentrations used were always 1 mM AgNO_3_ in 50 mM KNO_3_ (supporting electrolyte) except for the *ex situ* analysis, which omitted the supporting electrolyte.

### Macroscale electrochemical measurements

**Fig. 2:**
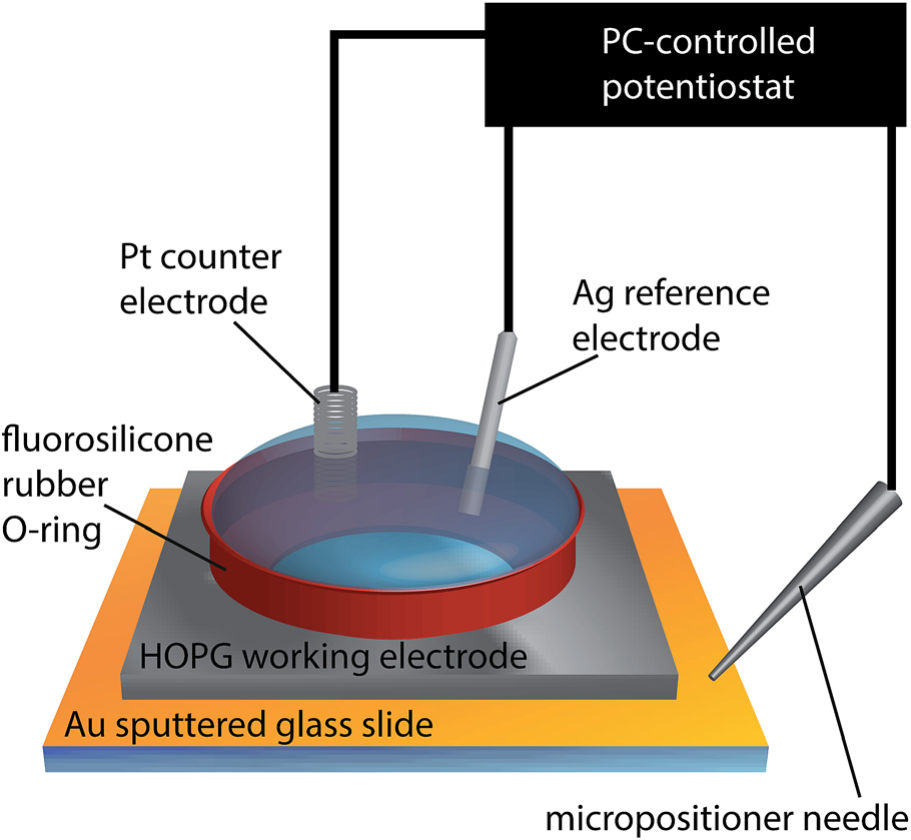
Schematic of the experimental set-up for macroscale electrochemical measurements.

Macroscopic electrochemical measurements were carried out in a droplet, confined by a 1/4 inch (6.4 mm) diameter fluorosilicone rubber O-ring gently placed on the HOPG surface, to produce a liquid-tight seal with little to no lateral friction or pressure-induced strain on the sample. A conventional three-electrode configuration was used, depicted in Fig. 2, where the HOPG substrate was connected as the working electrode, while a platinum gauze and a silver wire were used as counter and quasi-reference electrodes, respectively. Both the AM and SPI-3 HOPG samples were studied.

All potentials herein are reported relative to the Ag/Ag^+^ redox couple and can thus be directly related to the overpotential for the silver electrodeposition process. Depositions were performed from a solution of 1 mM AgNO_3_ in 50 mM KNO_3_, with the HOPG electrode held at –100, –170 and –240 mV. Prior to any deposition, immediately after cleaving and during/after solution addition, the surface was held at +400 mV for 180 s to avoid any deposition of silver. Deposition was induced by stepping the HOPG substrate to the potential of interest for defined periods up to 50 s, and recording the current–time response.

For experiments that were followed by *ex situ* microscopy characterisation (–100 mV driving force, see below), the deposition was carried out from a 1 mM AgNO_3_ solution without supporting electrolyte to minimize salt residues on the sample. This would result in some small difference in the applied driving force and mass transport rate, but broadly similar current–time curves (with inferred NP densities within a factor of 2) were recorded with and without supporting electrolyte. For microscopic analysis after deposition, the droplet was carefully removed using a Pasteur pipette, and any remaining solution whisked away using a fibre tissue placed at the edge of the sample. The macroscopic chronoamperometric measurements were performed at room temperature (21 ± 2 °C) in an air conditioned room, using a computer controlled CHI760A potentiostat (CH Instruments Inc., USA).

### Nanoscale measurements

**Fig. 3:**
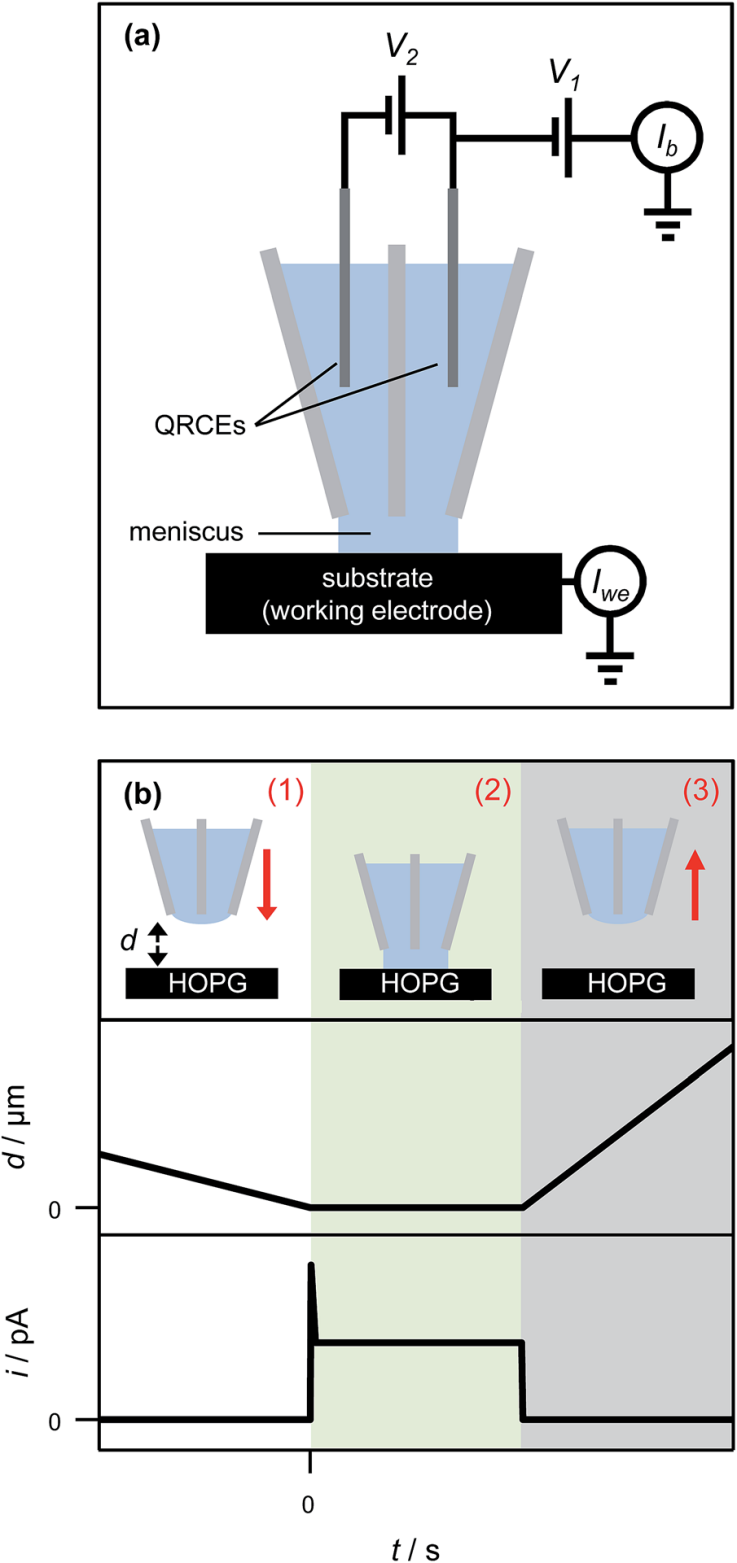
(a) Schematic of the experimental set-up for nanoscale electrochemical measurements. A potential bias (*V*_2_) was applied between the quasi-reference counter electrodes (QRCEs) before and after each measurement at a spot on the surface, and the current between them (*I*_b_) was measured to monitor the resistance of the electrolyte droplet. During electrodeposition experiments, no potential bias between the QRCEs was applied (*V*_2_ = 0); rather, both QRCEs were floated by potential *V*_1_ with respect to ground. The substrate (working electrode), held at ground, had an effective potential of –*V*_1_ with respect to the QRCEs, and the current flowing through it (*I*_we_) was measured continuously. (b) Schematic representation of pipette positioning during an electrodeposition experiment (top), together with the corresponding tip-to-substrate separation (middle) and current through the HOPG surface (bottom) as a function of time. (1) The pipette was translated slowly towards the HOPG surface. (2) Once the electrolyte droplet contacted the surface (as witnessed by a current spike), and assigned as *d* = 0, the pipette motion ceased automatically and the pipette was held in place for a predetermined time (typically 1 s). (3) The pipette was then retracted swiftly and moved laterally to approach at the next area. Red arrows denote the direction of movement of the pipette.

The SECCM set-up has been described in detail elsewhere^64^ and is shown schematically in Fig. 3(a). In short, a theta pipette was pulled to a sharp taper and filled with an electrolyte solution (1 mM AgNO_3_ in 50 mM KNO_3_), with one silver wire placed in each barrel as a quasi-reference counter electrode (QRCE). A small potential bias was applied between the QRCEs before and after each electrodeposition experiment to monitor the resistance of the electrolyte meniscus at the end of the pipette, which can be used to gauge the size of the droplet.^65^ This was used to verify that the size of the meniscus did not vary appreciably during the experiment, and also to minimize variances between experiments performed with different pipettes. There was no potential bias between the QRCEs during electrodeposition experiments.

The pipette was mounted above the HOPG sample, which was connected as the working electrode and held at ground. The driving potential in the cell was set by floating the potential of the QRCEs with respect to ground. Only AM grade HOPG was studied for the nanoscopic experiments, as it has the lowest number of step edge defects and is of the highest quality.^54,66^ The current flowing through the HOPG sample (working electrode current) was monitored continuously. The pipette was slowly lowered (200 nm s^–1^) towards the substrate, until a measureable current at the HOPG surface was detected, upon closing the electrical circuit, indicative of contact between the electrolyte droplet meniscus and the substrate, with no physical contact from the pipette itself. The meniscus was typically held on the surface for one second, while recording the current for Ag electrodeposition every 165 μs (the average of 32 separate measurements) on a home-built, high bandwidth current-to-voltage converter, before it was swiftly retracted (1 μm s^–1^) from the surface (Fig. 3(b)). The substrate was then moved laterally to provide a fresh HOPG area under the pipette, and the pipette was slowly lowered to bring the meniscus into contact again to perform another measurement. This entire procedure was typically repeated five times within *ca.* two minutes, and the measurements were found to be very reproducible (*vide infra*).

After localised electrodeposition, the HOPG surface was examined by field emission-scanning electron microscopy (FE-SEM). Also, to examine the contents of the capillary, the pipette was translated further into the surface after an electrodeposition, forcing the pipette to break and leave a minute droplet of solution and broken glass on the surface. A control tip breaking experiment was also performed, using only 50 mM KNO_3_ (*i.e.* in the absence of AgNO_3_) to ensure no electrodeposition. Both tip breaking experiments were inspected by FE-SEM, and electrodeposition features were also characterised by AFM. During electrodeposition, the electrochemical current response can be wholly assigned to the contacted area of the substrate, which is comparable to the pipette size (*ca.* 400 nm diameter).^53,59,65,67^

### *Ex situ* characterisation

FE-SEM images were recorded on a Zeiss Supra 55-VP. AFM images were recorded in tapping mode (TM-AFM) on a Veeco MultiMode AFM with a Nanoscope IIIa controller or a Veeco Enviroscope AFM with a Nanoscope IV controller.

## Results and discussion

### Silver electrodeposition on macroscopic HOPG surfaces

To investigate the role of step edge sites, we studied the electrodeposition of silver on two HOPG samples: AM grade and SPI-3 grade. The key difference between the two samples is the mean step density, which differs, on average, by a factor of 400 (Fig. 1).^58^ Thus, these samples are ideally suited to investigate the role of step edges in the electrodeposition of silver; any significantly higher reactivity at step edges sites compared to the basal plane sites would be expected to result in an enormous difference in kinetics, as reflected in the currents for the electrodeposition process. Surprisingly, while the key (often exclusive) role of step edges in the electrodeposition at HOPG has been reported in many papers,^50,68–71^ we are unaware of any previous work that has investigated the effect of step edge density by examining different grades of HOPG.

**Fig. 4:**
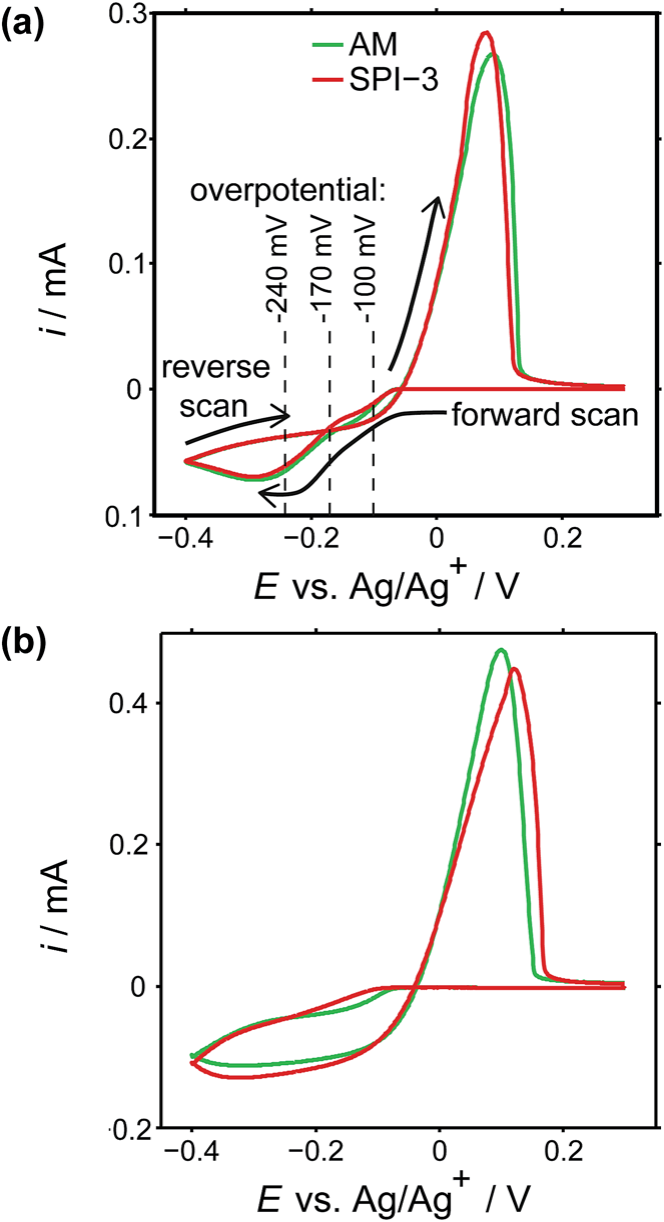
CVs of Ag electrodeposition and stripping (electrodissolution) on AM (green) and SPI-3 (red) HOPG, at (a) 100 mV s^–1^ and (b) 1 V s^–1^, using a macroscopic droplet cell. The horizontal lines signify several overpotentials at which current–time measurements were carried out.

Cyclic voltammograms (CVs) were recorded on both grades of HOPG, at 100 mV s^–1^ and 1 V s^–1^. The first voltammetric cycles for both surfaces are shown in Fig. 4, and display the characteristic signatures of metal electrodeposition on carbon surfaces.^28,45^ On the cathodic sweeps, a small nucleation overpotential (*ca.* 50 mV at 100 mV s^–1^, *ca.* 100 mV at 1 V s^–1^) is observed, leading to a characteristic peak that can be attributed to Ag electrodeposition. Reversal of the potential sweep direction gives rise to an anodic peak related to the Ag dissolution process. The total charge under the peaks is a measure of the amount of silver deposited or dissolved (depending on the sweep direction), and was the same for both processes. It can be seen that the CVs look virtually identical (peak currents, onset potentials and total charges) for both grades of HOPG, indicative of the same processes (thermodynamic and kinetic) occurring on both grades of HOPG at this timescale, even though the surface structure is hugely different (Fig. 1).

**Fig. 5:**
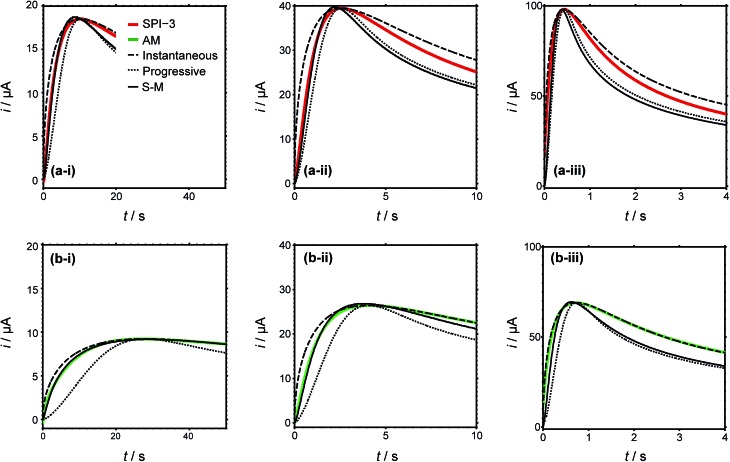
Current–time transients of silver electrodeposition in a macroscopic droplet cell on (a) SPI-3 (red lines) and (b) AM (green lines) HOPG at (i) –100 mV, (ii) –170 mV and (iii) –240 mV. Theoretical current–time transients predicted by the Scharifker–Hills models for the limiting cases of instantaneous (dashed) and progressive (dotted) nucleation and growth, as well as the best fit to the Sharifker–Mostany (S–M) model (solid).

Typical current–time transients for the electrodeposition of silver at different overpotentials indicated in Fig. 4(a) are shown in Fig. 5. The general morphology of these traces is that the current initially increases with time, representing the nucleation and growth of NPs that are (largely) diffusionally isolated, reaching a peak value followed by a decrease with time due to diffusional overlap (and, planar diffusion) of Ag^+^ to the resulting NP array. The peak moves to shorter time with increasing driving force, (i) to (iii), and the value is smaller (and slightly later) for AM grade HOPG. This tentatively suggests a smaller number of nucleating NPs on AM grade HOPG. Moreover, comparing the current–time transients of the two grades of HOPG side by side, the difference between them is not as significant as might be expected, if step edges were the exclusive nucleation and growth sites, based on the huge difference in step density. These observations thus strongly suggest that electrodeposition can occur to a significant extent on basal plane sites.

To further analyse the current–time behaviour, we have compared them to the Scharifker–Hills (S–H) models (see ESI, S1†) for the current–time transients, for both instantaneous and progressive nucleation.^30,50,72^ Models of this type are widely used to analyse chronoamperometric data for NP nucleation and growth.^72,73^ Importantly, it should be noted that these models are fully analytical and only require knowledge of the maximum current density and time at which this maximum occurs, *i.e.* there are no fitting parameters. It can be seen that particularly the rising part of the transients cannot be described adequately by the models for either instantaneous nucleation or progressive nucleation, instead showing nucleation behaviour intermediate between these two extreme cases, consistent with previous studies as discussed in the Introduction.^30,32–34^ More complex models have been developed,^74–76^ which allow the apparent number of nucleation sites on the surface to be obtained. In particular, Scharifker and Mostany derived the following general expression for the current for three-dimensional nucleation with diffusion controlled growth:

1 *i*(*t*)/*A* = (*nFc*(*D*/π*t*)^1/2^)(1 – exp{–*N*_0_π*D*(8π*cM*/*ρ*)^1/2^[*t*–(1 – exp(–*Bt*))/*B*]})

where *A* is the surface area of the electrode (0.322 cm^2^), *n* (=1) is the number of electrons in the redox process, *F* is the Faraday constant (*F* = 96 485 C mol^–1^), *c* is the bulk concentration (*c* = 1.0 × 10^–6^ mol cm^–3^), *D* is the diffusion coefficient (*D* = 1.5 × 10^–5^ cm^2^ s^–1^),^83^ *M* is the molar mass of the deposited metal (107.87 g mol^–1^), *ρ* is the density (10.49 g cm^–3^), *t* is the time (s), and *N*_0_ and *B* are the nucleation site density (in cm^–2^) and nucleation rate (in s^–1^), respectively. The least-squares fit of the experimental current–time transients to eqn (1) using *N*_0_ and *B* as fitting parameters are also shown in Fig. 5. While the results of the model provide a better match to the experimentally obtained data than the S–H models for the limiting cases (with no adjustable parameters), it captures the behaviour at longer times poorly. Additionally, discrepancies still remain in the rising part of the transient and the peak parameters, indicative of additional effects which are not described by a simple nucleation and growth model. Nonetheless, analyses of this type provide an estimate of the number of apparent nucleation sites (Table 1).

**Table 1:** Apparent number of nucleation sites extracted from the current–time transients for Ag electrodeposition on HOPG

Potential/mV	Estimated nucleation site density, *N*_0_/(10^5^ cm^–2^)	
	AM	SPI-3
–100	0.6	3.3
–170	5.8	28
–240	45	270

It can be seen that the apparent number of nucleation sites on SPI-3 HOPG is roughly five times that on AM, even though the step edge density is 400 times higher. Regardless of the quality of the HOPG (highly stepped compared to few steps), the apparent numbers of nucleation sites are within the range of 10^5^–10^7^ cm^–2^, for this range of driving forces, again consistent with previous findings from the analysis of chronoamperometric curves.^45,62^ This semi-quantitative analysis suggests a significant role of the basal surface in the NP nucleation and growth process.

**Fig. 6:**
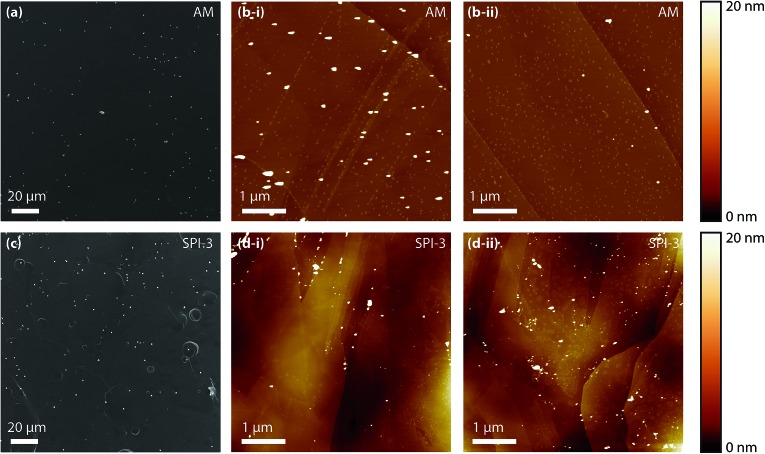
(a) FE-SEM image and (b-ii and b-ii) AFM images (two different areas) of macroscale electrodeposition of silver (from 1 mM AgNO_3_) on AM grade HOPG. (c) FE-SEM image and (d-i and d-ii) AFM images (two different areas) of macroscale electrodeposition on SPI-3 HOPG. The electrodeposition potential was –100 mV, held for 1 s.

To compare the apparent number of nucleation sites, derived from an analysis of the current–time transients, with the number of deposited particles, we characterised HOPG substrates after electrodeposition by FE-SEM and TM-AFM. It should be borne in mind that *ex situ* characterisation of metal NPs electrodeposited on HOPG can be complicated by the rather weak interaction between most metal NPs and the surface of sp^2^ carbon materials, particularly the HOPG basal plane (*vide infra*).^62,77–80^ Moreover, these measurements were made without KNO_3_ supporting electrolyte, as noted earlier, although this did not have a significant effect on the deposition transients. Thus, careful sample preparation and critical examination of the results from *ex situ* characterisation can provide powerful information on the electrodeposition process,^44,62,81^ and at least allows an estimate of the minimum number of NPs electrodeposited. Representative images are shown in Fig. 6.

The analysis of images, such as those shown in Fig. 6, is shown in Table 2. From the FE-SEM images (a and c), only particles larger than ∼100 nm (typically >∼1 μm) over a 370 μm^2^ area were counted due to the limited resolving power. Regions between such large particles were subsequently analysed by TM-AFM (b and d, 5 μm × 5 μm) to reveal many smaller particles. To distinguish between formed silver particles and residual salt crystals with AFM, control experiments were performed with a KNO_3_ solution without silver ions (see ESI, S2†), which showed that residual salt crystals are typically below 10 nm in size and located preferentially at the step edges. As such, only particles over 10 nm were included on the analysis in Table 2.

**Table 2:** Number of deposited particles from TM-AFM and FE-SEM analysis of macroscale deposition at –100 mV *vs.* Ag/Ag^+^

TM-AFM image analysis	AM	SPI-3
Particles/(10^8^ cm^–2^)	1.8 ± 1.1	3.6 ± 2.8
Particle size (height)/nm	24 ± 10	19 ± 13

FE-SEM image analysis	AM	SPI-3
Particles/(10^7^ cm^–2^)	5.0 ± 0.3	3.6 ± 0.5

This *ex situ* analysis showed that the number of electrodeposited particles was in the range of 10^7^–10^8^ cm^–2^, about three orders of magnitude higher than the number of apparent nucleation sites as obtained from analysis of the chronoamperometric transients (10^5^ cm^–2^ at the same driving force, *vide supra*). Moreover, within experimental error, there was no significant difference between the number of particles on AM and SPI-3 HOPG.

### Nanoscale silver deposition on HOPG using SECCM

Evidently, there is a considerable disparity between the number of silver particles derived from electrochemical data analysed with the S–H model and morphological analysis. Furthermore, a comparison of electrodeposition (current–time transients and microscopy) on AM and SPI-3 grade HOPG suggests a significant contribution of the basal surface to electrodeposition. Thus, to gain further insight into the process, we investigated silver deposition on AM grade HOPG with SECCM to elucidate the role of the HOPG basal plane, in isolation from step edge sites. We employed pipettes of *ca.* 400 nm diameter, thus limiting the effective working electrode area to the same dimensions. As AM grade HOPG typically provides surfaces with extensive basal planes (≫1 μm spacing between steps),^54^ this means that the contact area will typically only be the basal plane, with no step edges.^52^

While the initial rise in current for each event is too fast to consider in detail at the sampling rate employed, it provides important information on the number of nuclei that must be forming initially. The current for the electrodeposition of a single spherical particle is given by:^82^

2 *i*(*t*) = (2π*nF*(*Dc*)^3/2^*M*^1/2^*t*^1/2^)/*ρ*^1/2^

Eqn (2) yields a current of 1.6 pA for *t* = 200 μs, which is clearly much smaller than the values detected experimentally, indicating many nucleation processes in each event. These growing nuclei rapidly achieve diffusional overlap, leading to a diffusion-controlled growth regime (*vide infra*), *i.e.* quasi-linear diffusion of Ag^+^ ions down the barrels of the pipette, to the HOPG surface. The current decay occurs over the course of several milliseconds, allowing further analysis. In particular, Fig. 8, which is a zoom to a few characteristic events at the 3 potentials of interest, shows that the current decay for the individual current events at all potentials can be described by a modified Cottrell equation for a micro- or nanoelectrode:^84^

3 *i*(*t*) = nFAC*(*D*/π*t*)^1/2^ + nFAC*k_T_

where *A* is the effective surface area of the electrode (in cm^2^), and *k*_T_ is the steady-state mass transport coefficient (in cm s^–1^), which strongly depends on the geometry of the system. Eqn (3) assumes the reaction to be driven at the maximum rate, which is reasonable for applied overpotentials of –100 mV and –200 mV, but will work less well for –50 mV. Nonetheless it provides a reasonable approach for the semiquantitative interpretation of the electrochemical data.

**Fig. 7:**
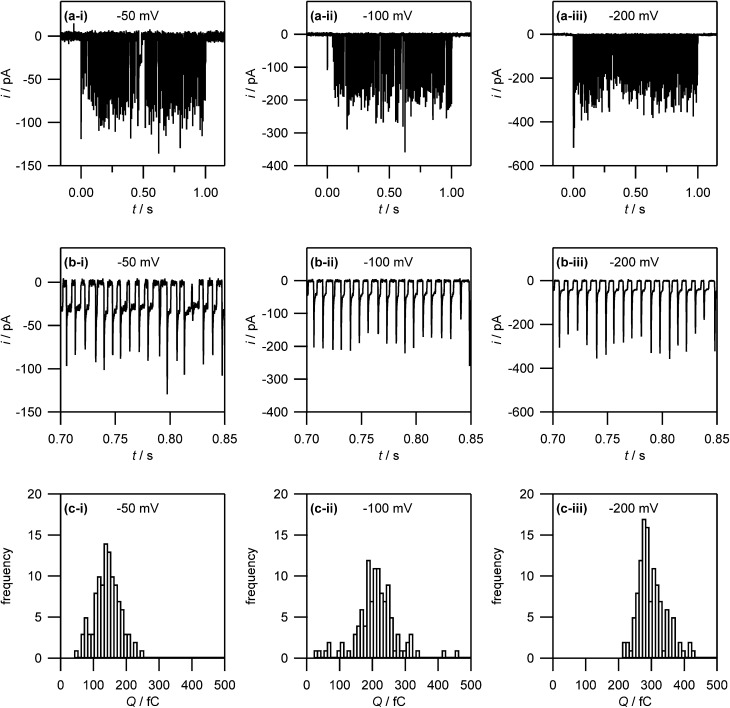
(a) Current–time traces for the electrodeposition of silver (from 1 mM AgNO_3_ in 50 mM KNO_3_) on HOPG at –50 mV, –100 mV and –200 mV. Note that no electrodeposition takes place before *t* = 0 s and after *t* = 1 s as the electrolyte droplet is not in contact with the substrate. (b) Zoom-in on the current–time traces in (a) to show the discrete current events. (c) Histogram of the charges associated with the discrete current events.

**Fig. 8:**
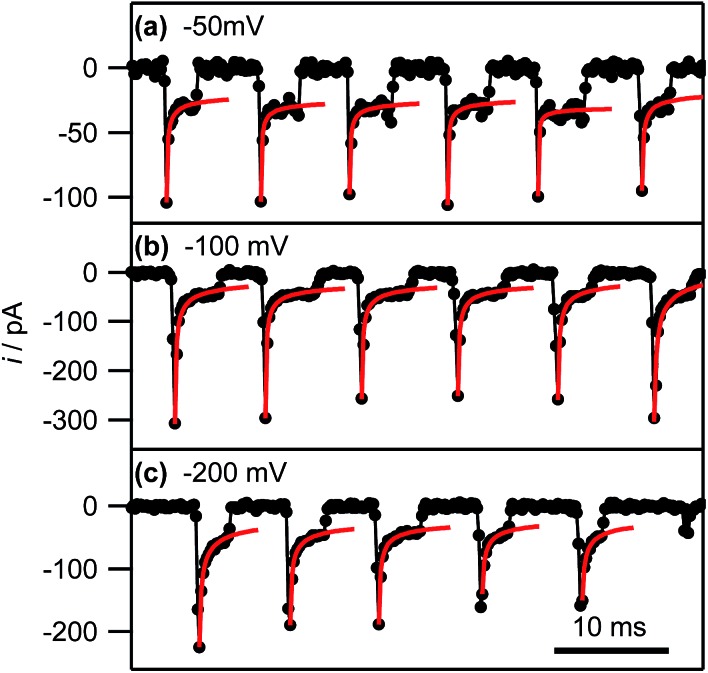
Experimental current–time traces (black connected circles) and fits of the individual current events to eqn (3) (red lines; see main text) at the indicated potentials. The full range of the horizontal axis corresponds to 50 ms.

By fitting all current events to eqn (3), using the *A* and *k*_T_ as the two fitting parameters, we obtain *A* = 3.2 ± 1.3 × 10^–9^ cm^2^ (corresponding to a disk electrode with radius of *ca.* 300 nm) and *k*_T_ = 0.05 ± 0.02 cm s^–1^ for electrodeposition at –50 mV. The fits of the current events at –100 mV and –200 mV reveal broadly similar values (see ESI, S4†). The obtained values for *A* are in reasonable agreement with the pipette size, further supporting the idea that the growth of the NPs after the initial current peak is governed by quasi-linear diffusion down the pipette towards the HOPG surface (*vide infra*). Similarly, the steady-state mass transfer coefficient is an order of magnitude lower than for an inlaid disk electrode of the same radius (*k*_T,disk_ = 4*D*/π*r*, where *r* is the disk radius),^84^ which we have found to be the typical magnitude for (sub-)micrometre sized pipettes with cone angles *ca.* 8–10° as used herein.^52,65^

Interestingly, after a few ms, the current for a particular event ceases rather abruptly. This is assigned to the detachment of Ag from the surface. There is then a small induction time before the next current event (*vide infra*).

**Fig. 9:**
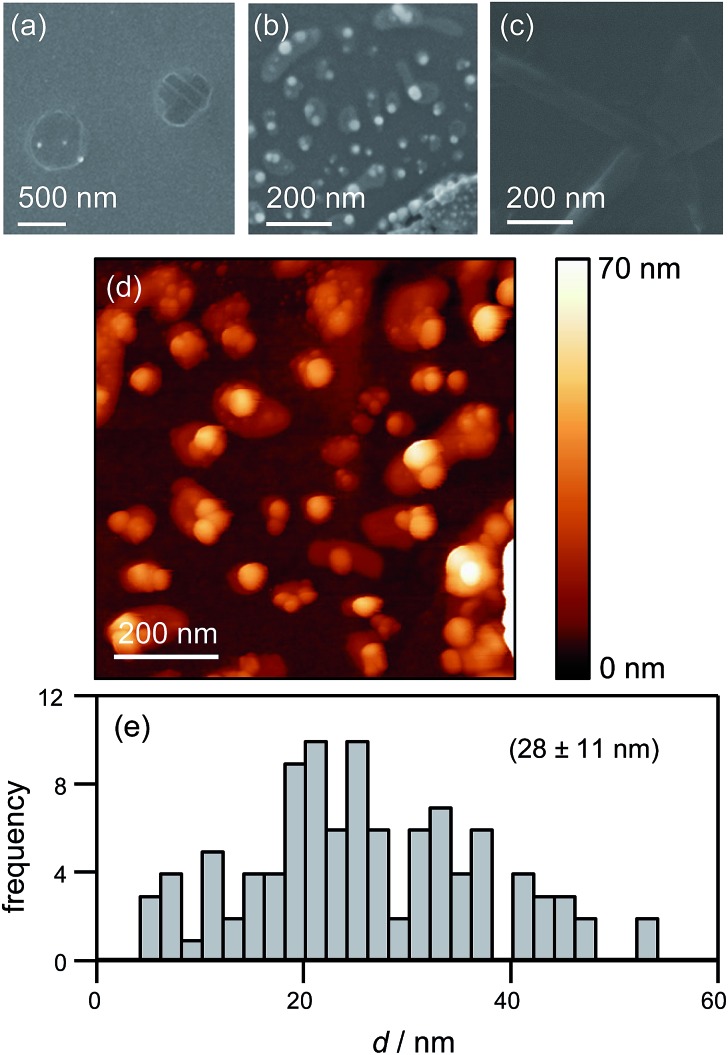
HOPG surface visualization after electrodeposition. (a) FE-SEM image of two deposition spots. (b) FE-SEM image of a deposition spot after controlled breaking of the pipette. Some glass from the pipette is visible in the lower right corner. (c) FE-SEM image of a controlled pipette breaking, without Ag deposition. (d) TM-AFM image of the same region as studied in (b). (e) Histogram of NP size obtained from the TM-AFM image in (d). The electrodeposition potential (for all data except (c)) was –50 mV.

To corroborate NP formation, we examined the HOPG surface after an electrochemical experiment performed at –50 mV with high resolution microscopy (Fig. 9).

Fig. 9(a) shows an FE-SEM image of the HOPG substrate after electrodeposition experiments. Two spots are clearly visible where the electrolyte meniscus was brought into contact with the HOPG surface, and electrochemical measurements were made. These features further substantiate that the contact area is comparable to the area of the pipette opening (*ca.* 400 nm diameter). Notably, even though the current–time traces associated with each of these spots showed the typical behaviour outlined above (*ca.* 100–150 discrete current events during the contact time of 1 s), only a few NPs can be observed. This is consistent with our proposed mechanism: NPs detach quickly after their formation and are transported into the electrolyte solution in the pipette. By retracting the pipette after the deposition period, the electrolyte with the particles in solution is withdrawn from the surface, leaving a mostly clean surface.

To confirm NP formation, a small amount of electrolyte was forced from the pipette to the surface after an electrodeposition experiment by slightly lowering the pipette onto the HOPG surface, thereby leaving behind a minute drop of AgNP-containing electrolyte solution on the HOPG surface. Fig. 9(b) shows a FE-SEM image of the NPs deposited this way, where part of a piece of glass from the pipette can be seen in the bottom-right corner. Comparing Fig. 9(b) with (a), it can clearly be seen that breaking the pipette leaves a large number of NPs of *ca.* 30–40 nm diameter on the surface. These findings clearly indicate a large number of NPs in the electrolyte solution in the pipette after an electrodeposition experiment. As these NPs are pristine (*i.e.* not capped by stabilizing agents), and of controlled size, we envisage that this method could be exploited as an approach for NP synthesis. In addition, it may be possible to fine-tune the mean particle size by varying the silver salt and the supporting electrolyte concentration, the substrate electrode, the temperature and the potential field across the electrolyte meniscus by applying a bias potential between the two QRCEs.

A pipette breaking control experiment was also performed, *i.e.* a pipette filled with just supporting electrolyte (at the same concentration) was forced onto the surface, to examine the tip contents, while eliminating the effect of electrodeposition. The resulting surface is devoid of nanoparticles (Fig. 9(c)), although there are traces of salt. An enlarged view of the broken pipette on the surface is shown in ESI, S5.†

The same area of the HOPG surface where electrodeposition followed by tip breaking was carried out, was also investigated with TM-AFM. A typical TM-AFM image is shown in Fig. 9(d). While the background is somewhat noisy, possibly due to some salt residues from the electrolyte solution and carbon deposition from prior FE-SEM imaging, the NPs are clearly visible. A histogram of NP heights estimated from the TM-AFM image is shown in Fig. 9(e), which reveals a size distribution of 28 ± 11 nm, which appears consistent with the charge histogram in Fig. 7(c).

It should, however, be mentioned that the TM-AFM size distribution is complicated by a number of issues. First, as discussed above, the background in the TM-AFM image is somewhat noisy, making it difficult to distinguish between smaller particles (<10 nm) and background features. Consequently, the occurrence of NPs with a size below 10 nm is most likely overestimated, and the frequencies in the histogram for particles <10 nm can be considered an upper limit. Furthermore, a closer inspection of Fig. 9(b) and (d) shows that the NPs are often agglomerated. This agglomeration is most likely induced by the drying of the electrolyte droplet. Naturally, agglomerates will have a larger apparent height, skewing the distribution towards larger NPs. Finally, some degree of Ostwald ripening can occur for AgNPs in solution after they detach from the substrate,^37,85,86^ facilitated by the presence of silver ions in solution. This would further widen the size distribution. Although these factors complicate full quantitative comparison between the electrochemical (current–time) results and the data from high-resolution microscopy, the important point is that the findings from electrochemical measurements and microscopy are consistent with a nucleation-aggregative growth-detachment mechanism for the formation of multiple AgNPs on an HOPG electrode.

**Fig. 10:**
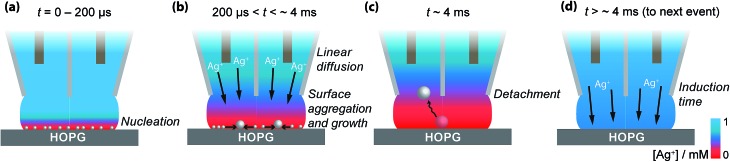
Schematic representation of the nucleation-aggregative growth-detachment mechanism, where *t* is the time. After the AgNPs nucleate (a), the growing NPs quickly consumes all of the Ag^+^ in the electrolyte meniscus to reach a state where further growth of NPs is limited by diffusion of Ag^+^ down the barrels of the pipette, during which time NPs can aggregate on the HOPG surface (b). This happens until the AgNPs reach a critical size/total charge and detach from the surface, shown here for a solitary particle at which time the surface concentration of Ag^+^ is 0 mM (c). After detachment, the surface concentration of Ag^+^ is replenished by diffusion down the barrels of the pipette (d), at which point the process can repeat with another nucleation event (a).

Based on these findings the time-dependency of the nucleation-aggregative growth-detachment mechanism for Ag electrodeposition on HOPG is summarized in Fig. 10. Initially, many small critical nuclei are formed at the surface in a concatenated event (Fig. 10(a)). As the nuclei form they rapidly (<200 μs) achieve a diffusion-controlled growth regime by consuming all of the silver ions at the electrode surface (Fig. 10(a)), and further growth of the NP is limited by the quasi-linear diffusion of silver ions down barrels of the pipette into the electrolyte meniscus (Fig. 10(b)), in agreement with the Cottrell analysis outlined above. At the surface, small mobile silver nuclei aggregate into clusters in a process that lowers their surface tension (Fig. 10(b)). This aspect supports the view that electrochemical deposition follows an aggregative growth pathway, when considered on the nanoscale, as shown recently by Ustarroz *et al.* ^87,88^ Finally, as the NPs reach a critical cluster size after 3–5 ms, and surface tension is sufficiently low, the particle(s) detach from the surface, and are transported into the electrolyte solution (Fig. 10(c)).

Before the nucleation-aggregative growth-detachment cycle can restart (Fig. 10(a)), the zone needs to be replenished by Ag^+^ ions diffusing back to the surface (Fig. 10(d)). This diffusion of Ag^+^ ions results in a short induction time of a few ms (Fig. 10(d)). For a typical SECCM pipette tip of the type used herein, the diffusional time, *t*_diff_, follows the relationship^53,65^

4 *t*_diff_ ≈ (10*r*)^2^/*D*

The diffusional time is *ca.* 3 ms when *a* = 200 nm, as for this study, consistent with the induction time between transient events.

Induction times for metal electrodeposition on (arrays of) nanoelectrodes are well known,^38,89^ and may be related to the adsorption and lateral movement of a few individual Ag atoms on the surface to form a critical nucleus at each nucleation site for the next growth event.^47,90^ This analysis is consistent with the induction time constant having a narrow distribution (see ESI, S3,† for histograms of induction times), and not changing markedly with overpotential.

### General discussion

The proposed nucleation-aggregative growth-detachment mechanism obviously merits some discussion, particularly in view of previous literature on metal (and, in particular, silver) electrodeposition on HOPG (highlighted above and considered further herein). In most studies, it has often been suggested that the active sites for metal deposition are the step edges and that the atomically smooth basal plane needs to be activated (by some pre-treatment to introduce atomic scale defects)^29,47,91^ for metal nucleation and deposition to occur. It should, however, be kept in mind that these findings are typically based on *ex situ* characterisation of the deposited particles, which introduce additional artifacts due to sample preparation or characterisation techniques. For example, it has been shown that in scanning probe microscopy methods, such as AFM and scanning tunneling microscopy, NPs can be displaced or dislodged by the probe.^44,92,93^ Similarly, further sample preparation after electrodeposition experiments such as removing the electrolyte solution, rinsing the surface, and drying the surface can involve forces which are sufficient to overcome the weak metal–HOPG interaction and alter the NP size (due to agglomeration) and spatial distribution on the HOPG surface. As such, the finding that NPs are preferentially located at step edges from *ex situ* characterisation does not necessarily identify the active sites for metal nucleation and growth; instead, it indicates that step sites act as ‘anchoring’ sites for metal NPs, *i.e.* sites where the metal–substrate interaction is sufficiently strong for a NP to remain stuck, either due to geometric effects or local surface functionalities.

In this context, closer examination of some previous work reveals that significant electrodeposition of NPs can occur on the HOPG basal plane,^28,44,62,94–96^ particularly where care was taken to minimize the lateral forces on the NPs during sample preparation and characterisation, precautions which we took in our studies. Our findings that metal nucleation can occur readily on the HOPG basal plane, through both macroscale and nanoscale measurements, can be interpreted similarly.

Silver is known to be somewhat mobile on HOPG,^79,80^ and, as a result, growing nuclei and NPs will move around on the HOPG surface until hitting an ‘anchoring’ site, primarily step edges. This provides some explanation for the apparent faster growth rates on SPI-3 compared to AM grade HOPG (Fig. 1), although the higher specific surface area of SPI-3 HOPG will also be a small factor. The average density of metal nucleation sites on HOPG has been reported in the range of 10^6^–10^10^ cm^–2^, depending on the analysis performed.^29,44,45,62,81,94,95^ As such, given that the contact area in our SECCM is of the order of the pipette diameter (*ca.* 400 nm), Fig. 9(a), the number of point defects on the HOPG substrate would be very limited (and, interestingly, could be zero).^51,97–99^ Given the size of the pipettes employed compared to the average step spacing of the HOPG substrate, the contacted region of the substrate would typically only consist of the HOPG basal plane with no step sites. Consequently, there is no ‘anchoring’ site for the AgNP, which keeps growing until the entropic gain of the NP being free in solution is greater than the interaction energy between the NP and the substrate, and the NP detaches from the surface and diffuses into the electrolyte solution. We can rule out NP detachment due to an electric field between the pipette barrels, as no potential bias was applied between the QRCEs (Fig. 3(a)), as often used in SECCM,^65^ and the supporting electrolyte concentration was high (50 mM). It is also interesting to note that the electrodeposition of metal nanoparticles,^100–103^ and particularly Ag NPs,^82,104^ occurs readily at liquid/liquid interfaces, often with little overpotential required. These are obviously defect free interfaces, and provide a precedent for aspects of the mechanism proposed herein.

The nucleation-aggregative growth-detachment mechanism also sheds light on the discrepancy between the observed behaviour of macroscopic silver electrodeposition with the S–H model, which shows behaviour that lies between the instantaneous and progressive nucleation and growth models. Furthermore, at –100 mV applied potential, the number of nucleation sites predicted by the analytical model at low driving force (10^5^ cm^–2^) is significantly lower than the number of particles observed using SEM (10^7^ cm^–2^) and AFM (10^8^–10^9^ cm^–2^). The SEM analysis counted particles over 100 nm, while the AFM images counted particles smaller than this, over ∼10 nm. Both observations are consistent with and can be rationalized by the proposed nucleation-aggregative growth-detachment mechanism. As electrodeposition is initiated, nucleation and growth will occur at each nucleation site. The formed NPs are mobile due to the weak interaction between Ag and HOPG. As the nucleation sites are freed up due to the NPs mobility, further nucleation can occur, leading to a single nucleation site producing many NPs. The NP mobility encourages electrochemical aggregative growth, as shown by Ustarroz *et al.* ^87,88^ This work on aggregative growth, together with our results on macroscopic nucleation, complements the nucleation-aggregative growth-detachment mechanism proposed for SECCM, considering that the S–H analysis is, in this case, too simplistic to take account of detachment events and surface mobility. A significant point about the size and geometry of the SECCM meniscus set-up is that the particles are encouraged to detach from the surface (high volume/surface ratio) and since the contact area is so small that aggregated particles cannot grow too large and the barrels provide a route of exodus for mobile particles that break away from the weak surface interaction.

A further indication for the detachment of the NPs during growth (rather than, for example, when retracting the pipette) is the total number of current events. As shown in Fig. 7, the number of current events, and thus the minimum number of NPs formed during 1 s is *ca.* 100–150. Experiments carried out over longer times showed that current events were maintained over the course of (at least) several minutes (see ESI, S6†). With an average NP diameter of 30 nm, there would simply not be enough space on the contacted area of the substrate (*ca.* 400 nm diameter) to accommodate all of the NPs formed if the NPs remained on the surface.

## Conclusions

In conclusion, the studies in this paper reveal important new aspects and complexities to metal electrodeposition at solid electrode surfaces, through investigations at a range of length scales and time scales. Using an SECCM-based approach it has been shown that silver electronucleation and growth on the basal surface of HOPG at the nanoscale is a non-continuous process. Owing to the timescale and length scale of SECCM, we have been able to probe the HOPG basal plane in isolation of HOPG step edges with current–time measurements, allowing us to resolve the nucleation and growth of NPs on the HOPG basal plane. Interestingly, it has been found that the electrodeposition of silver on HOPG follows a nucleation-aggregative growth-detachment mechanism. This finding has been further supported by macroscale measurements, where there is a significant disparity between the number of calculated nucleation sites (events) from chronoamperometry, and the number of particles observed by high resolution microscopy. The macroscale and nanoscale techniques probe different parts of the HOPG surface and by comparing samples of different quality (step edge density) it was shown that step edge sites did not contribute significantly to the number nucleation events and that the basal plane was a key location for nucleation.

Interestingly, under the SECCM experimental conditions, the AgNPs grow to *ca.* 30 nm before detaching from the surface and diffusing into the solution opening up a new route to the tailored synthesis of a few NPs. In addition to opening up new prospects for the study of individual NP electrodeposition, the studies herein reveal key features and a model that sheds new light on the understanding of metal electrodeposition processes on carbon electrodes, in general. The data herein also add to a growing body of evidence on the intrinsic electroactivity of the basal surface of HOPG, showing that it can support fast rates of electron transfer for a wide range of electrochemical processes.

## References

[cit1] Sun C. Q., Tay B. K., Zeng X. T., Li S., Chen T. P., Zhou J., Bai H. L., Jiang E. Y. (2002). J. Phys.: Condens. Matter.

[cit2] Zanchet D., Tolentino H., Martins Alves M. C., Alves O. L., Ugarte D. (2000). Chem. Phys. Lett..

[cit3] Li H., Han P. D., Zhang X. B., Li M. (2013). Mater. Chem. Phys..

[cit4] Haruta M. (2003). Chem. Rec..

[cit5] Koper M. T. M. (2011). Nanoscale.

[cit6] Li Y., Boone E., El-Sayed M. A. (2002). Langmuir.

[cit7] Link S., El-Sayed M. A. (1999). J. Phys. Chem. B.

[cit8] Fu H. B., Yao J. N. (2001). J. Am. Chem. Soc..

[cit9] Hernández-Santos D., González-García M. B., García A. C. (2002). Electroanalysis.

[cit10] Anker J. N., Hall W. P., Lyandres O., Shah N. C., Zhao J., Van Duyne R. P. (2008). Nat. Mater..

[cit11] Nie S. M., Emery S. R. (1997). Science.

[cit12] Kneipp K., Kneipp H., Itzkan I., Dasari R. R., Feld M. S. (1999). Chem. Rev..

[cit13] Bard A. J. (2010). J. Am. Chem. Soc..

[cit14] Arvia A. J., Salvarezza R. C., Triaca W. E. (2004). J. New Mater. Electrochem. Syst..

[cit15] Kitsomboonloha R., Ngambenjawong C., Mohammed W. S., Chaudhari M. B., Hornyak G. L., Dutta J. (2011). Micro Nano Lett..

[cit16] Pankhurst Q. A., Connolly J., Jones S. K., Dobson J. (2003). J. Phys. D: Appl. Phys..

[cit17] Moghimi S. M., Hunter A. C., Murray J. C. (2001). Pharmacol. Rev..

[cit18] Chithrani B. D., Ghazani A. A., Chan W. C. W. (2006). Nano Lett..

[cit19] Lokina S., Stephen A., Kaviyarasan V., Arulvasu C., Narayanan V. (2014). Eur. J. Med. Chem..

[cit20] Prabhu S., Poulose E. (2012). Int. Nano Lett..

[cit21] Masuda H., Yasui K., Nishio K. (2000). Adv. Mater..

[cit22] Howells A. R., Hung L., Chottiner G. S., Scherson D. A. (2002). Solid State Ionics.

[cit23] Yeung K. L., Wolf E. E. (1992). J. Vac. Sci. Technol., A.

[cit24] Henry C. R. (1998). Surf. Sci. Rep..

[cit25] Haynes C. L., Van Duyne R. P. (2001). J. Phys. Chem. B.

[cit26] Turkevich J., Stevenson P. C., Hillier J. (1951). Discuss. Faraday Soc..

[cit27] Brust M., Walker M., Bethell D., Schiffrin D. J., Whyman R. (1994). J. Chem. Soc., Chem. Commun..

[cit28] Penner R. M. (2002). J. Phys. Chem. B.

[cit29] Bayati M., Abad J. M., Nichols R. J., Schiffrin D. J. (2010). J. Phys. Chem. C.

[cit30] Scharifker B., Hills G. (1983). Electrochim. Acta.

[cit31] Tian N., Zhou Z.-Y., Sun S.-G., Ding Y., Wang Z. L. (2007). Science.

[cit32] Gunawardena G., Hills G., Montenegro I., Scharifker B. (1982). J. Electroanal. Chem..

[cit33] Hills G., Pour A. K., Scharifker B. (1983). Electrochim. Acta.

[cit34] Gunawardena G., Hills G., Montenegro I., Scharifker B. (1982). J. Electroanal. Chem..

[cit35] Garcia-Pastoriza E., Mostany J., Scharifker B. R. (1998). J. Electroanal. Chem..

[cit36] Dudin P. V., Unwin P. R., Macpherson J. V. (2010). J. Phys. Chem. C.

[cit37] Redmond P. L., Hallock A. J., Brus L. E. (2004). Nano Lett..

[cit38] Chen S., Kucernak A. (2003). J. Phys. Chem. B.

[cit39] Tel-Vered R., Bard A. J. (2006). J. Phys. Chem. B.

[cit40] Velmurugan J., Noël J.-M., Nogala W., Mirkin M. V. (2012). Chem. Sci..

[cit41] Cox J. T., Zhang B. (2012). Annu. Rev. Anal. Chem..

[cit42] Ebejer N., Schnippering M., Colburn A. W., Edwards M. A., Unwin P. R. (2010). Anal. Chem..

[cit43] Ebejer N., Güell A. G., Lai S. C. S., McKelvey K., Snowden M. E., Unwin P. R. (2013). Annu. Rev. Anal. Chem..

[cit44] Zoval J. V., Stiger R. M., Biernacki P. R., Penner R. M. (1996). J. Phys. Chem..

[cit45] Miranda-Hernandez M., Gonzalez I., Batina N. (2001). J. Phys. Chem. B.

[cit46] Porter J. D., Robinson T. O. (1993). J. Phys. Chem..

[cit47] Pötzschke R. T., Gervasi C. A., Vinzelberg S., Staikov G., Lorenz W. J. (1995). Electrochim. Acta.

[cit48] McCreery R. L., McDermott M. T. (2012). Anal. Chem..

[cit49] Desai D., Turney D. E., Anantharaman B., Steingart D. A., Banerjee S. (2014). J. Phys. Chem. C.

[cit50] Endres F., Freyland W., Gilbert B. (1997). Ber. Bunsen-Ges..

[cit51] Boxley C. J., White H. S., Lister T. E., Pinhero P. J. (2002). J. Phys. Chem. B.

[cit52] Williams C. G., Edwards M. A., Colley A. L., Macpherson J. V., Unwin P. R. (2009). Anal. Chem..

[cit53] Lai S. C. S., Patel A. N., McKelvey K., Unwin P. R. (2012). Angew. Chem., Int. Ed..

[cit54] Patel A. N., Collignon M. G., O'Connell M. A., Hung W. O. Y., McKelvey K., Macpherson J. V., Unwin P. R. (2012). J. Am. Chem. Soc..

[cit55] Anne A., Cambril E., Chovin A., Demaille C., Goyer C. (2009). ACS Nano.

[cit56] Lhenry S., Leroux Y. R., Hapiot P. (2012). Anal. Chem..

[cit57] Kirkman P. M., Güell A. G., Cuharuc A. S., Unwin P. R. (2013). J. Am. Chem. Soc..

[cit58] Patel A. N., Tan S.-y., Miller T. S., Macpherson J. V., Unwin P. R. (2013). Anal. Chem..

[cit59] Patel A. N., McKelvey K., Unwin P. R. (2012). J. Am. Chem. Soc..

[cit60] Edwards M. A., Bertoncello P., Unwin P. R. (2009). J. Phys. Chem. C.

[cit61] Lee C.-Y., Guo S.-X., Bond A. M., Oldham K. B. (2008). J. Electroanal. Chem..

[cit62] Gloaguen F., Leger J. M., Lamy C., Marmann A., Stimming U., Vogel R. (1999). Electrochim. Acta.

[cit63] Patel A. N., Tan S.-y., Unwin P. R. (2013). Chem. Commun..

[cit64] Kleijn S. E. F., Lai S. C. S., Miller T. S., Yanson A. I., Koper M. T. M., Unwin P. R. (2012). J. Am. Chem. Soc..

[cit65] Snowden M. E., Güell A. G., Lai S. C. S., McKelvey K., Ebejer N., O'Connell M. A., Colburn A. W., Unwin P. R. (2012). Anal. Chem..

[cit66] Zhang G., Kirkman P. M., Patel A. N., Cuharuc A. S., McKelvey K., Unwin P. R. (2014). J. Am. Chem. Soc..

[cit67] Güell A. G., Ebejer N., Snowden M. E., McKelvey K., Macpherson J. V., Unwin P. R. (2012). Proc. Natl. Acad. Sci. U. S. A..

[cit68] Walter E. C., Murray B. J., Favier F., Kaltenpoth G., Grunze M., Penner R. M. (2002). J. Phys. Chem. B.

[cit69] Walter E. C., Zach M. P., Favier F., Murray B. J., Inazu K., Hemminger J. C., Penner R. M. (2003). ChemPhysChem.

[cit70] Favier F., Walter E. C., Zach M. P., Benter T., Penner R. M. (2001). Science.

[cit71] Vazquez L., Hernandez Creus A., Carro P., Ocon P., Herrasti P., Palacio C., Vara J. M., Salvarezza R. C., Arvia A. J. (1992). J. Phys. Chem..

[cit72] Tsakova V., Milchev A. (1987). J. Electroanal. Chem..

[cit73] Lu G., Zangari G. (2005). J. Phys. Chem. B.

[cit74] Scharifker B. R., Mostany J. (1984). J. Electroanal. Chem..

[cit75] Heerman L., Tarallo A. (2000). Electrochem. Commun..

[cit76] Sluyters-Rehbach M., Wijenberg J. H. O. J., Bosco E., Sluyters J. H. (1987). J. Electroanal. Chem..

[cit77] Hula R. C., Edtmaier C., Holzweber M., Hutter H., Eisenmenger-Sittner C. (2010). Appl. Surf. Sci..

[cit78] He Y., Zhang J. Y., Wang Y., Yu Z. P. (2010). Appl. Phys. Lett..

[cit79] Goldby I. M., Kuipers L., von Issendorff B., Palmer R. E. (1996). Appl. Phys. Lett..

[cit80] Couillard M., Pratontep S., Palmer R. E. (2003). Appl. Phys. Lett..

[cit81] Liu H., Favier F., Ng K., Zach M. P., Penner R. M. (2001). Electrochim. Acta.

[cit82] Guo J., Tokimoto T., Othman R., Unwin P. R. (2003). Electrochem. Commun..

[cit83] Johnston R. R. M., Spiro M. (1967). J. Phys. Chem..

[cit84] Bard A. J. and Faulkner L. R., Electrochemical Methods: Fundamentals and Applications, 2nd edn, 2001.

[cit85] Silvert P. Y., HerreraUrbina R., Duvauchelle N., Vijayakrishnan V., Elhsissen K. T. (1996). J. Mater. Chem..

[cit86] Bastus N. G., Comenge J., Puntes V. (2011). Langmuir.

[cit87] Ustarroz J., Ke X., Hubin A., Bals S., Terryn H. (2011). J. Phys. Chem. C.

[cit88] Ustarroz J., Hammons J. A., Altantzis T., Hubin A., Bals S., Terryn H. (2013). J. Am. Chem. Soc..

[cit89] Quinn B. M., Lemay S. G. (2006). Adv. Mater..

[cit90] Ustarroz J., Gupta U., Hubin A., Bals S., Terryn H. (2010). Electrochem. Commun..

[cit91] Hendricks S. A., Kim Y. T., Bard A. J. (1992). J. Electrochem. Soc..

[cit92] Schaefer D. M., Patil A., Andres R. P., Reifenberger R. (1993). Appl. Phys. Lett..

[cit93] Schaefer D. M., Ramachandra A., Andres R. P., Reifenberger R. (1993). Z. Phys. D: At., Mol. Clusters.

[cit94] Patten H. V., Ventosa E., Colina A., Ruiz V., López-Palacios J., Wain A. J., Lai S. C. S., Macpherson J. V., Unwin P. R. (2011). J. Solid State Electrochem..

[cit95] Gimeno Y., Creus A. H., Carro P., Gonzalez S., Salvarezza R. C., Arvia A. J. (2002). J. Phys. Chem. B.

[cit96] Brülle T., Stimming U. (2009). J. Electroanal. Chem..

[cit97] Obeng Y. S., Bard A. J. (1991). J. Am. Chem. Soc..

[cit98] Ma H., Lee L., Brooksby P. A., Brown S. A., Fraser S. J., Gordon K. C., Leroux Y. R., Hapiot P., Downard A. J. (2014). J. Phys. Chem. C.

[cit99] Stevenson K. J., Veneman P. A., Gearba R. I., Mueller K. M., Holliday B. J., Ohta T., Chan C. K. (2014). Faraday Discuss..

[cit100] Izquierdo D., Martinez A., Heras A., Lopez-Palacios J., Ruiz V., Dryfe R. A. W., Colina A. (2012). Anal. Chem..

[cit101] Uehara A., Hashimoto T., Dryfe R. A. W. (2014). Electrochim. Acta.

[cit102] Johans C., Kontturi K., Schiffrin D. J. (2002). J. Electroanal. Chem..

[cit103] Grunder Y., Ho H. L. T., Mosselmans J. F. W., Schroeder S. L. M., Dryfe R. A. W. (2011). Phys. Chem. Chem. Phys..

[cit104] Li F., Edwards M., Guo J., Unwin P. R. (2009). J. Phys. Chem. C.

