# Supplementary material for: Nucleation, aggregative growth and detachment of metal nanoparticles during electrodeposition at electrode surfaces
Source: Chem Sci. 2014 Nov 7;6(2):1126–38. doi: 10.1039/c4sc02792b (PMC5811076; doi:10.1039/c4sc02792b)
Supplement: Supplementary file 1 [file SC-006-C4SC02792B-s001.pdf]

Electronic Supplementary Material (ESI) for Chemical Science.
This journal is © The Royal Society of Chemistry 2014

Supplementary Information for

Nucleation, aggregative growth and detachment of metal nanoparticles during electrodeposition at electrode surfaces

Stanley C. S. Lai,[1,2,*] Robert A. Lazenby,[1] Paul M. Kirkman,[1] and Patrick R. Unwin[1,*]

[1] Department of Chemistry, University of Warwick, Gibbet Hill Road, Coventry CV4 7AL, UK

[2] MESA+ Institute for Nanotechnology, University of Twente, PO Box 217, 7500 AE Enschede, The Netherlands

[*] To whom correspondence should be addressed: s.c.s.lai@utwente.nl; p.r.unwin@warwick.ac.uk

S1 Scharifker-Hills model

Scharifker and Hills (S-H) modelled the nucleation and growth current-time transients for multiple nuclei, in the limiting cases of instantaneous and progressive nucleation and growth.[1] These processes are conveniently analysed by simple analytical expressions, requiring only the magnitude and the time of the maximum current density. Instantaneous nucleation and growth is described by

$$\frac{I^2}{I_m^2} = \frac{1.9542}{t/\ t_m}\left\{1 - \exp\left[-1.2564(t/\ t_m)\right]\right\}^2 \quad \text{\textbackslash* MERGEFORMAT (S.1)}$$

where I is the current density, I_m is the maximum current density, t is time and t_m is the time at the maximum current. Progressive nucleation can be expressed as

$$\frac{I^2}{I_m^2} = \frac{1.2254}{t/\ t_m}\left\{1 - \exp\left[-2.3367(t/\ t_m)^2\right]\right\}^2 . \quad \text{\textbackslash* MERGEFORMAT (S.2)}$$

Experimental current-time transients shown in Figure 4 (main text) were analysed using these expressions using experimentally obtained values for I_m and t_m.

[1] B. Scharifker and G. Hills, *Electrochim. Acta*, 1983, 28, 879-889.

S2 Tapping mode-atomic force microscopy (TM-AFM) image of AM grade HOPG, after exposure to a droplet of 50 mM KNO_3

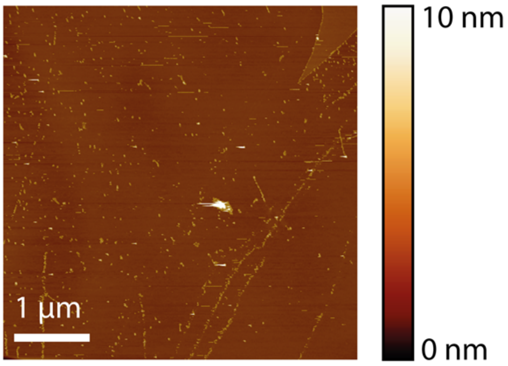

The macroscale deposition experiments were mimicked using a solution with just KNO_3 present (*i.e.*, no Ag^+). The droplet was removed, as described in the main text, and the surface was imaged by TM-AFM. Features can be observed on the surface, below the 10 nm threshold, which can be attributed to residual salt crystals. No features above 10 nm were observed.

S3 Distribution of induction times for Ag deposition with SECCM

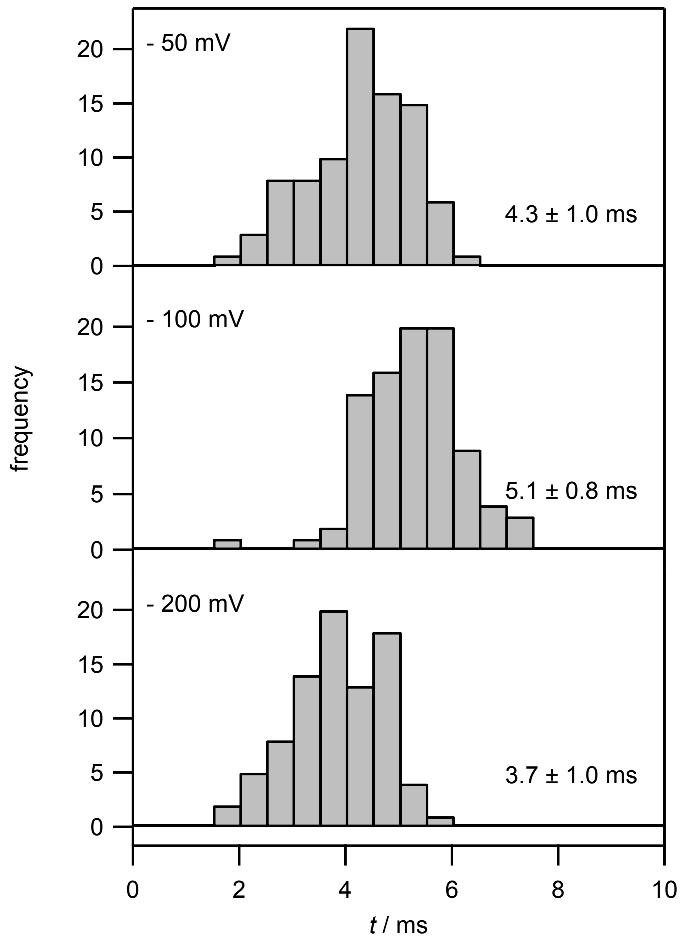

Histograms of induction times between distinct current events at the various electrodeposition potentials are shown above and discussed in the main text.

S4 Results of the modified Cottrell fits of the current-time transients obtained with SECCM at different potentials

	-50 mV	-100 mV	-200 mV	Average over all potentials
Area (/10^{-9} cm^2)	3.2 ± 1.3	6.9 ± 2.8	7.4 ± 3.3	5.6 ± 3.2
Corresponding radius (/nm)	320 ± 65	470 ± 95	485 ± 110	420 ± 120
k_T (/10^{-2} cm s^{-1})	4.8 ± 2.4	3.6 ± 2.7	3.7 ± 1.9	3.9 ± 2.6

S5 FE-SEM images of HOPG after controlled SECCM tip breaking

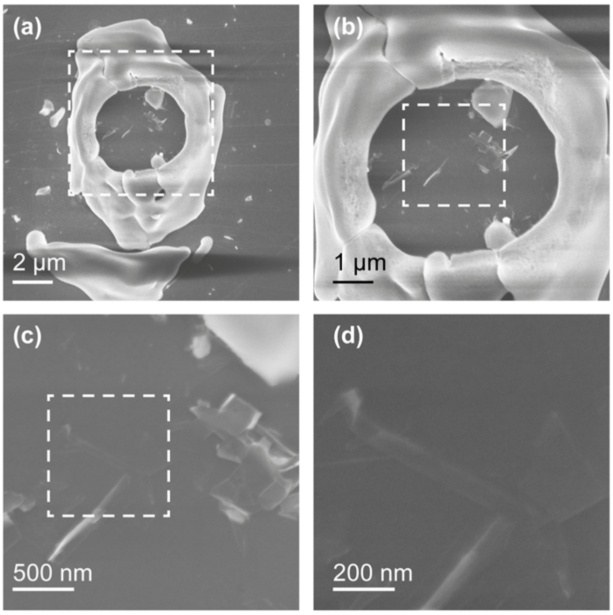

FE-SEM images of the site on HOPG of a controlled breaking of the pipette, which contained 50 mM KNO_3 (*i.e.* no Ag^+), at various magnifications. (a) The entire site of the broken pipette. (b) The central cavity of the tip. (c) The HOPG surface at the site of pipette breaking. (d) The surface, as depicted in Figure 9(c) in the main text. Areas of successive magnification are highlighted with dashed lines. Note that there are some effects of insulating material (glass and salt) charging in (a) and (b). Also, fragments of the septum of the theta capillary are visible in (a) and (b).

S6 Extended current-time trace for Ag deposition with SECCM

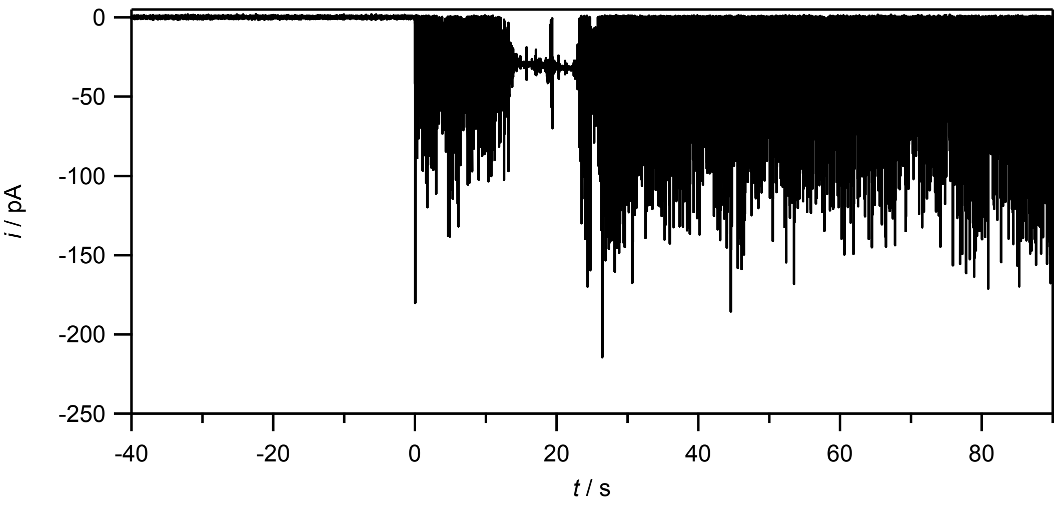

Current-time trace for the electrodeposition of silver (from 1 mM $AgNO_3$ in 50 mM KNO_3) on HOPG at − 200 mV. No electrodeposition takes place before $t = 0$ s as the electrolyte droplet is not in contact with the substrate. Here, one data point was collected every 6.4 ms (average of 256 measurements every 25 μs) to limit the total amount of data collected over the longer time period. As a consequence, the time per data point here is comparable to the total length of one nucleation-growth-detachment cycle (see main text). Thus, the lack of observable individual events between 15 and 25 s is likely due to the convolution of individual events.
